# The Chinese-born immigrant infant feeding and growth hypothesis

**DOI:** 10.1186/s12889-016-3677-6

**Published:** 2016-10-11

**Authors:** Kristy A. Bolton, Peter Kremer, Kylie D. Hesketh, Rachel Laws, Karen J. Campbell

**Affiliations:** 1 Institute for Physical Activity and Nutrition (IPAN), School of Exercise and Nutrition Sciences, Deakin University, Geelong, Victoria Australia; 2 School of Exercise and Nutrition Sciences, Deakin University, Geelong, Victoria Australia

**Keywords:** Infant, Rapid growth, Feeding practices, Chinese, Australia, Immigrants, Culture, Overweight, Obesity, Maternal child health

## Abstract

**Background:**

Rapid growth in the first six months of life is a well-established risk factor for childhood obesity, and child feeding practices (supplementation or substitution of breast milk with formula and early introduction of solids) have been reported to predict this. The third largest immigrant group in Australia originate from China. Case-studies reported from Victorian Maternal and Child Health nurses suggest that rapid growth trajectories in the infants of Chinese parents is common place. Furthermore, these nurses report that high value is placed by this client group on rapid growth and a fatter child; that rates of breastfeeding are low and overfeeding of infant formula is high. There are currently no studies which describe infant growth or its correlates among this immigrant group.

**Presentation of hypothesis:**

We postulate that in Australia, Chinese-born immigrant mothers will have different infant feeding practices compared to non-immigrant mothers and this will result in different growth trajectories and risk of overweight. We present the Chinese-born immigrant infant feeding and growth hypothesis - that less breastfeeding, high formula feeding and early introduction of solids in infants of Chinese-born immigrant mothers living in Australia will result in a high protein intake and subsequent rapid growth trajectory and increased risk of overweight and obesity.

**Testing the hypothesis:**

Three related studies will be conducted to investigate the hypothesis. These will include two quantitative studies (one cross-sectional, one longitudinal) and a qualitative study. The quantitative studies will investigate differences in feeding practices in Chinese-born immigrant compared to non-immigrant mothers and infants; and the growth trajectories over the first 3.5 years of life. The qualitative study will provide more in-depth understanding of the influencing factors on feeding practices in Chinese-born immigrant mothers.

**Implications of the hypothesis:**

This study will provide evidence of the potential modifiable feeding practices and risk of overweight faced by Chinese-born immigrants living in Australia. This is important to help identify groups at risk of rapid growth and subsequent risk of obesity, to identify opportunities for intervention, and to be able to tailor prevention initiatives appropriately.

## Background

Childhood overweight and obesity is a major public health concern, with 25.1 % of Australian children aged 2–17 years estimated to be overweight (18.2 %) or obese (6.9 %) [1]. Rapid growth in the first six months of life is a well-established risk factor for childhood obesity [2–4], and early childhood feeding practices (supplementation or substitution of breast milk with formula and early introduction of solids) have been reported to predict this [5–9]. Other important predictors of rapid weight gain during infancy include maternal factors such as pre-pregnancy body mass index (BMI), excess gestational weight gain and smoking whilst pregnant [2]. Understanding growth trajectories and correlates of growth in our infant populations is important to identify opportunities for obesity prevention early in life.

Australia has a multicultural population of over 24.1 million [10]. In 2014, an estimated 28.1 % of the population was born overseas - with China being the third highest immigrant group (1.9 % of the population) [11]. Immigrant groups are an important focus for obesity prevention as evidence suggests that during acculturation to high income countries, many immigrants will adopt adiposity promoting behaviours [12–15]. Case-studies from Victorian Maternal and Child Health (MCH) nurses suggest that rapid growth trajectories in the infants of Chinese-born immigrant mothers is common place. Further, MCH nurses report this client group to place high value on rapid growth and a fatter child; low breastfeeding rates and overfeeding with infant formula. It is important we are responsive to this evidence from MCH nurses, who are key providers of early childhood healthcare in Australia.

In the absence of information regarding rapid growth of infants of Chinese-born immigrants residing in Australia, this paper draws upon the available evidence to present a hypothesis for rapid growth trajectories and subsequent elevated risk of overweight and obesity in infants of Chinese-born immigrants. Studies to test the hypothesis will be described. This knowledge will identify opportunities for intervention and inform the development of prevention strategies to ensure that these initiatives are tailored appropriately.

## Presentation of the hypothesis

### The Chinese-born immigrant infant feeding and growth hypothesis

**Fig. 1:**
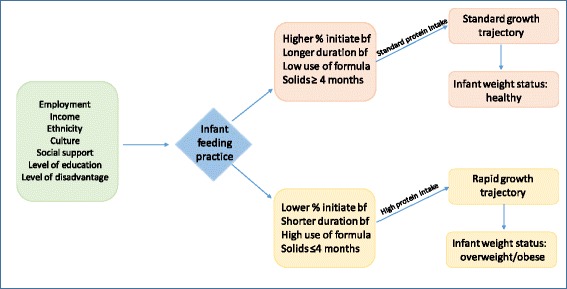
The Chinese-born immigrant infant feeding and growth hypothesis Based upon case studies from MCH nurses, we hypothesise that infants of Chinese-born immigrant mothers will expose their infants to less breastfeeding, more infant formula feeding, earlier introduction of solid foods – all which will increase protein intake and subsequently result in a rapid growth trajectory and risk of overweight and obesity in early childhood (by 3.5 years old). %: proportion of mothers; bf: breastfeeding. Note: analysis of data to explore the Chinese-born immigrant infant feeding and growth hypothesis will adjust for age, parity, maternal BMI, smoking status

We propose the Chinese-born immigrant infant feeding and growth hypothesis - compared to infants of non-immigrant mothers living in Australia, infants of Chinese-born immigrant mothers will be exposed to less breastfeeding, more infant formula feeding, and earlier introduction of solid foods (all which increase infant protein intake). We further hypothesise that these exposures will increase the risk of: rapid growth in the first year of life, overweight and obesity during infancy and early childhood (Fig. 1). Components of the hypothesis postulated to influence infant feeding and growth are described below.

#### Ethnicity

Whilst childhood obesity is increasing in all ethnic groups; the prevalence of obesity is higher in non-white populations and influenced by genetics, physiology, culture, socioeconomic status, environment as well as interactions between these variables [16]. Ethnic background has been demonstrated to be an important risk factor for overweight and obesity in Australian primary-school aged children [17–19]. However, there is limited evidence on children with Chinese backgrounds and this needs further investigation.

#### Cultural factors

Cultural factors such as language, religion, health beliefs, values and behaviours are suggested to influence child and adult obesity [18]. For example, the Chinese cultural belief that a fat baby is a healthy baby [20, 21] may foster feeding practices that promote rapid weight gain and consequently increased risk of childhood overweight and obesity. The cultural practice of grandparents living with and being the key provider of childcare to their grandchildren in China has also been shown to influence the risk of childhood obesity through their attitudes, indulging behaviours and poor health knowledge [22].

#### Feeding practices, rapid weight gain and risk of overweight and obesity

##### Breastfeeding

The initiation and maintenance of breastfeeding may explain differential growth trajectories, however the evidence is contradictory. Evidence suggests breastfeeding to be protective against rapid growth [23] and childhood obesity [5, 6], though not all studies have adjusted for confounding factors [24] known to impact child growth (e.g. maternal factors such as socioeconomic status, weight status, smoking, birth weight) [25]. Nevertheless, studies controlling for maternal obesity, smoking and socioeconomic status have still demonstrated significant associations, although the size of the association was reduced [25]. A recent meta-analysis revealed a 10 % reduction in the prevalence of overweight or obesity in children exposed to a longer duration of breastfeeding after adjustment for social factors [26].

The rates of breastfeeding in Chinese living in Australia are unclear. One study reports 6 % of Chinese-Australian mothers to exclusively breastfeed until six months [27], with lower breastfeeding intentions and initiation rates compared to Australian English-speaking mothers [28]. Asian women in Australia have been found to be more likely to be partially breastfeeding (other liquid/solids are given in addition to breastmilk) at six weeks and 12 weeks postpartum compared to non-Asians [29]. Factors suggested to influence breastfeeding practices in Chinese immigrant women include: cultural (spiritual beliefs, the “hot and cold” theory whereby rituals and consumption of particular foods aim to restore balance in the body postpartum), the role of family (spouse, grandparents, particularly the paternal grandmother), Chinese cultural interventions (e.g. language appropriate education) and the surrounding community [30]. There is also a Chinese cultural misconception that Australian infant formula is better-quality than that in China [20].

##### Formula feeding

High protein formula feeding may contribute to the risk for overweight and obesity [31, 32]. Compared with human breastmilk, infant formula has different macronutrient composition (high energy, high protein, low fat), hormonal and microflora components which may affect infant growth rates, appetite, energy utilisation and eating behaviours [33]. Koletzko et.al proposed the Early Protein Hypothesis - infants with high protein consumption (in metabolic excess) will have early weight gain, adipogenic activity and long-term obesity risk [5, 34]. Accelerated weight gain due to formula feeding in a longitudinal study following Australian infants until 20 years old was suggested to be due to an upward BMI centile crossing on growth charts [23]. Rate of growth is considered important because of associations with later overweight and obesity [2–4].

##### Feeding practices and introduction of solids

The current recommendation for introduction of solids is at around six months [35]. Whilst not conclusive, emerging evidence from a systematic review suggests very early introduction of complementary foods before four months, may increase the risk of a child being overweight [7]. However this association has not been finitely confirmed due to the complex nature of overweight and obesity, and inconsistencies in capturing and adjusting for confounding variables within individual studies. The timing of introducing solids to infants of immigrant Chinese in Australia is unknown.

#### Other factors

The importance of confounding factors (i.e. family and socioeconomic variables) [4] which are risk factors for obesity cannot be underestimated. Whilst not central to the hypothesis, factors such as infant birth weight, maternal weight (pre- and post- pregnancy), maternal smoking, parity, age, socio-economic status, income, employment, level of education, family/social support, marital status, need to be controlled for.

### Testing the hypothesis – the Chinese-born immigrant infant feeding and growth studies

#### Study 1 – Infant feeding practices of Chinese-born immigrant mothers living in Australia – a cross-sectional quantitative study utilising the Australian National Infant Feeding Survey

The Australian National Infant Feeding Survey (2010) was a large scale, national survey of infant feeding practices in infants 0–24 months old (*n* = 28,759) [36]. This de-identified dataset will be analysed to assess potential differences in infant feeding practices between Chinese-born immigrant mothers (*n* = 602) and a randomly selected subsample of non-immigrant mothers (*n* = 602) living in Australia. Infant feeding practices that will be analysed include prevalence rates of breastfeeding and infant formula feeding, the age of exposure to other liquids (e.g. water, cow’s milk, toddler milk, soy milk, water-based drinks, fruit juice); and solids. Statistical analysis of this data will include descriptive statistics (means and standard deviations, or proportions; with differences between ethnic groups and infant feeding practices and demographic characteristics determined by Chi-square tests or t-tests as appropriate) and multiple linear regression to examine the influence of ethnicity on the age that liquids and solids were first introduced.

#### Study 2 – Weight trajectories and infant feeding practices of Chinese-born immigrant mothers living in Australia – a longitudinal quantitative study

The MCH service in Victoria, Australia provides free pre-scheduled appointments from birth until school age [37]. Anthropometric and feeding-related data are collected and enable nurses to track children’s health and growth from birth to school age. A longitudinal analysis (from birth to 3.5 years old) of growth trajectories using a de-identified database from a Local Government Area in Victoria (Australia) with a high proportion of Chinese-born immigrants (7 % of population [38]) will be conducted to examine potential differences in growth trajectories; in addition to breastfeeding, formula feeding and timing of the introduction of solids between infants of Chinese-born immigrants (*n* = 934) compared with a random subsample of non-immigrants (*n* = 934). Zscores (bmi-for-age (zbmi), weight-for-age (zwei), length/height-for-age (zlen)) will be calculated using WHO growth standards [39] and rapid weight gain will be defined as an increase (≥0.67) in zbmi from birth to 12 months [40, 41]. Descriptive statistics will be conducted (means and standard deviations, or proportions; with differences between ethnic groups and mean zscores, rapid weight gain and demographic characteristics determined by Chi-square tests or t-tests as appropriate). Growth curve modelling techniques will be conducted to examine the growth trajectories over time.

#### Study 3 – Qualitative description of feeding practices

The aim of this study is to investigate the Chinese mother’s beliefs, their attitudes to breastfeeding and formula feeding, introduction of solids to gain an understanding of the influencing factors on feeding practices and infant growth. Chinese-born immigrant mothers will be recruited for the study from an area in Melbourne which has a high population of Chinese immigrants. Individual semi-structured interviews (*n* ~ 36) will be recorded, transcribed and an inductive thematic analysis will be conducted.

### Implications of the hypothesis

We propose the Chinese-born infant feeding and growth hypothesis. This study will examine the impact of early feeding practices and identify potentially modifiable risk factors for rapid weight gain and subsequent later risk of overweight. Outcomes from this study will inform future prevention opportunities and allow them to be tailored specifically for the Chinese immigrant subgroup.

## Conclusion

Case studies suggest rapid weight gain in infants of immigrant Chinese in Australia. There are currently no data to confirm these reports. Given that this ethnic group is the third largest immigrant population in Australia [11], understanding growth trajectories and risk factors for rapid weight gain and subsequent childhood overweight and obesity is crucial in order to identify potentially modifiable targets for obesity prevention. The outcomes of this study will contribute to identifying immigrant groups at risk of rapid growth and subsequent risk of obesity; will identify opportunities for intervention, and will inform appropriate tailoring of prevention initiatives to this immigrant group.

## Abbreviations

BMI: Body mass index
MCH: Maternal child health
